# Apple varieties, diseases, and distinguishing between fresh and rotten through deep learning approaches

**DOI:** 10.1371/journal.pone.0322586

**Published:** 2025-05-15

**Authors:** Tao Zhang, Mustafa Mhamed, Qu Zhang, Liling Yang, Z.H.A.O. Xiaohui, Gu Haiyan, Zhao Zhang

**Affiliations:** 1 Department of Materials and Architectural Engineering, Hebei Institute of Mechanical and Electrical Technology, Xingtai, China; 2 Key Laboratory of Smart Agriculture System Integration, Ministry of Education, Beijing, China; 3 Key Laboratory of Agricultural Information Acquisition Technology, Ministry of Agriculture and Rural Affairs, China Agricultural University, Beijing, China; 4 College of Information and Electrical Engineering, China Agricultural University, Beijing, China; 5 Research Institute of Agricultural Mechanization, Xinjiang Academy of Agricultural Sciences, Urumqi, China; 6 International Office, China Agricultural University, Beijing, China; 7 Information Engineering College, Shandong Business Institute, Yantai, Shandong, China; Nanchang University, CHINA

## Abstract

Apples are one of the most productive fruits in the world, in addition to their nutritional and health advantages for humans. Even with the continuous development of AI in agriculture in general and apples in particular, automated systems continue to encounter challenges identifying rotten fruit and variations within the same apple category, as well as similarity in type, color, and shape of different fruit varieties. These issues, in addition to apple diseases, substantially impact the economy, productivity, and marketing quality. In this paper, we first provide a novel comprehensive collection named Apple Fruit Varieties Collection (AFVC) with 29,750 images through 85 classes. Second, we distinguish fresh and rotten apples with Apple Fruit Quality Categorization (AFQC), which has 2,320 photos. Third, an Apple Diseases Extensive Collection (ADEC), comprised of 2,976 images with seven classes, was offered. Fourth, following the state of the art, we develop an Optimized Apple Orchard Model (OAOM) with a new loss function named measured focal cross-entropy (MFCE), which assists in improving the proposed model’s efficiency. The proposed OAOM gives the highest performance for apple varieties identification with AFVC; accuracy was 93.85%. For the apples rotten recognition with AFQC, accuracy was 98.28%. For the identification of the diseases via ADEC, it was 99.66%. OAOM works with high efficiency and outperforms the baselines. The suggested technique boosts apple system automation with numerous duties and outstanding effectiveness. This research benefits the growth of apple’s robotic vision, development policies, automatic sorting systems, and decision-making enhancement.

## 1. Introduction

The apple is now grown in the majority of nations, and as it has the most significant cultivar variation among fruit-bearing trees, it can be grown in both the warmest and coldest climates on Earth [1,2]. China is among the world’s top apple producers and the most popular fruits eaten worldwide [3]. The development of the inputs in the manufacturing sector, such as weed protection preparations, mineral fertilizer, agricultural mechanization, packaging materials, and the building of storage facilities, is greatly influenced by advancements in the field of fruit production in general, including the growing of apples [4–9].

The emergence of the artificial intelligence revolution has given birth to a variety of platforms for activities centered on apples [10], such as pruning [11], pollination [12,13], harvesting [14–17], sorting [18,19], and more [20,21], benefiting both economics and productivity [22,23].

The developments are separated into two groups: one for apparatus [14,24–27] and devices [25,28,29] and another related to the vision [30–36]. Every advancement in one area raises the bar and boosts productivity in the others, improving overall quality and efficiency.

In agricultural automation, automated fruit sorting and categorization are essential [14,37]. With robotics systems, automatic fruit classification methods may be utilized during harvest to identify different varieties of fruits and separate them for harvesting [18]. These may also be used for fruit harvesting, pricing identification in supermarkets for expedited billing, and post-harvest quality testing for the packing sector. To increase fruit quality and production, attempts have been made to replace labour-intensive manual fruit picking and sorting procedures with automated systems that use machine vision and machine learning methods [28].

Several deep learning and machine learning strategies have driven recent advancements in apple fruit vision. We will start with the apple fruit varieties via machine learning and deep learning techniques and conclude with apple disease prediction.

An assessment comparing the Naive Bayes (NB) approach to PCA, Fuzzy Logic, and MLP-Neural methods for categorization was published in [38]. The results showed that the NB performed comparably to PCA, Fuzzy Logic, and MLP-Neural with 91.00%, 90.00%, 89.00%, and 83.00%, respectively. This study showed that NB has a high degree of assurance when accurately classifying apple varieties.

A novel fuzzy clustering technique called fuzzy discriminant c-means (FDCM) clustering was introduced in [39] to classify apple varieties using near-infrared spectroscopy. Near-infrared reflectance (NIR) spectra were obtained in the investigations using 200 samples of four different apple kinds. PCA was used to reduce the data’s excessive dimensionality. 97.00% was the highest score that was obtained. To distinguish between excellent and bad apples, the authors of [40] used logistic regression, Support Vector Machines (SVM), and k-NN. The targeted characteristics were the Law’s Texture Energy (LTE), Tamura features, Gray level co-occurrence matrix (GLCM), and histogram of oriented gradients (HOG). SVM scored top on average, performing at 98.90%. Six different apple varieties were evaluated, and an identification approach was provided by Bhargava et al. [41]: Granny Smith, Golden Delicious, Fuji, York, Jonagold, and Red Delicious. Use grab-cut and fuzzy c-means clustering for the picture segmentation. Afterwards, principal component analysis (PCA) is used to extract the features. Lastly, k-NN, SVM, LR, and SRC classifiers differentiate between rotten and fresh apples. At 95.27%, the SVM achieved the highest score. Stefany et al. [42] provided a multivariate discriminating analytical evaluation for apple ripening with a precision of 91.05%.

Regarding deep learning methods, we can observe that Shruthi et al. [43] presented a convolutional neural network (CNN) architecture and evaluated it against the most advanced designs, ResNet50, VGG16, MobileNet, and EfficientNetB0; the proposed CNN can attain an improved accuracy of 99.02% when identifying 14 different kinds of apples. The transfer learning approach was updated and assessed by Silverio et al. [44] on a database that had nine kinds of apple varieties to classify various Asturian apple varieties. InceptionV3 scored the highest overall accuracy of (98.04%). Feng et al. [45] developed a CNN-based system for identifying and categorizing apple quality, and it underwent testing on datasets containing 300 apples. An overall score was 95.33%.

In [38], the NB score was 91.00%, which improved the automated sorting system. [39] performance up to 97.00%, but the evaluation was done on a view sample with four apple types. In [40], SVM successfully classified rotten and fresh with 98.90% accuracy. With 95.27%, SVM has the highest efficiency, but it has six apple varieties this time. [43] used CNN on 14 apple kinds, and the score was 99.02%, for [44] Inception gave 98.04% in nine apple types. For Apple quilty classification, CNN’s best accuracy was 95.33%.

Based on the above results, most machine learning models utilized are NB, SVM, and RF. These have limitations when handling vast amounts of data, besides needing additional units to improve the performance. For Deep learning, CNN algorithms are used for apple varieties [39,43–45]. The minimum number of apple types evaluated was four [39], and the maximum was fourteen [43]. Only [40,41] assessed to categorize among the fresh and rotten apples.

Now, we will overview the works related to apple diseases. Several image processing approaches have been used to build automated diagnostic systems for apple disease. These solutions provide an alternative to farmers’ time-consuming and expensive conventional techniques, such as visual symptoms by eye diagnostics [46,47].

Concerning the apple disease part, several investigations have been conducted using diverse machine and deep learning methodologies. Dubey et al. [48] used two models for the detection and classification of apple fruit diseases in datasets containing 431 images, with four classes: apple scab, rot, blotch, and normal. The first K-means for the segmentation process, then extracting features, and a multi-SVM for the classification had an accuracy of 93.00%. In [49], they utilized a K-means-based approach to identify diseased parts using MSVM as a multi-class classifier to detect apple fruit diseases automatically. This research validates the procedure by validating 320 samples of three apple diseases—blotch, rot, and scab—and healthy apples. The research findings demonstrate that the suggested approach can accurately classify apple-related illnesses up to 95.94%. In [50], they suggested a method to classify apple fruit illnesses such as apple blotch, apple scab, and apple rot. First, stem and calyx areas are identified through thresholding-based segmentation, and the segmented picture is then enhanced using morphological techniques. Secondly, faulty areas are refined using a masking technique and a K-means clustering-based defect segmentation approach. After that, the segmented apple photos extract characteristics based on color, texture, and form. They applied classification-bagging decision trees, and the best result was up to 96.00%. Turkoglu et al. [51] presented multimodal LSTM-based pre-trained convolution neural networks (MLP-CNNs) as an ensemble majority voting method on 1192 apple pictures to identify plant illnesses and pests. The highest accuracy rating was 99.20%. In [52], they proposed five models based on CNN for recognising apple disease on 3200 images with three classes: apple scab, blotch, and rot. Model 5 had the best performance, up to 99.17%. In [53], they investigated different Deep CNN (DCNN) applications (ResNet, SqeezeNet, and MiniVGGNet) to boost apple disease detection. The dataset consists of 319 (i.e., 80 images for healthy, blotch, and rot apple images, respectively, and 79 for Scab) images of apples. The highest performance of DCNN is up to 98.00% compared to the baseline. Sugiarti et al. [54] combined the GLCM for extraction function with an NB approach and tested on 391 images with four classes: apple blotch (104), apple rot (107), apple scab (100), and regular apples (80). The classification accuracy was 96.43%.

Recently, Azgomi et al. [55] developed an ADD-NN method for categorizing pictures of apples with fungal infections and for neural network-based illness diagnosis. A k-means algorithm for the color features was used as an extra duty to separate the fruit’s damaged from healthy regions. Following the features’ extraction, they were designated as the inputs of a multi-layer perceptron neural network, with the four diseases—scab, bitter rot, black rot, and healthy fruits—being the network’s four outputs. The procedure’s efficiency using various architectures for the neural network trained with 60% of the data was then evaluated. The best accuracy was 73.70%.

For machine learning, [48,49] applied SVM on three apple disease types: scale, rot, and blotch; accuracy was 93.00% and 95.94%, respectively. The training was done on less than 500 samples and, in [50], used a bagging decision Tree with 96.00% efficiency. For deep learning, [51] employed Lstm based on (MLP-CNN) with excellent performance up to 99.20%. [52,53] used CNN with 99.17% and 98.00%, respectively. GLCM was tested in [54] and performed at 96.43%; for [55], ADD-NN was used with 73.70% accuracy. It should be noted that most research, whether using deep learning or computers, only investigated three categories of apple diseases: scab, rot, and blotch. Furthermore, the models needed to provide comprehensive estimates of illnesses and were trained on a smaller volume of data.

We will now provide an overall overview based on the relevant works that was previously discussed. Concerning machine learning techniques, we see that SVM was most often used to categorize apple varieties and had the best performance compared to other algorithms. Consuming a long training duration for big datasets. Understanding and interpreting the final visualization, particular influence, and varying weights may be challenging because the model unities are limited to minor model measurements, which makes sophisticated optimizations difficult to implement. The CNN architecture for deep learning was the most widely used and showed superior performance. The best approach for making predictions in apple disease studies was the multi-modal LSTM-based pre-trained convolution neural networks (MLP-CNNs). The most popular model in segmentation was the K-mean. All the models worked well compared to previous traditional systems. However, some issues are still related to executing high computations and deep local feature extractions, especially when handling massive datasets and extensive and overlapped classes. Therefore, it is essential to implement advanced technologies in the development process, which will improve performance, speed, and quality.

**Fig 1:**
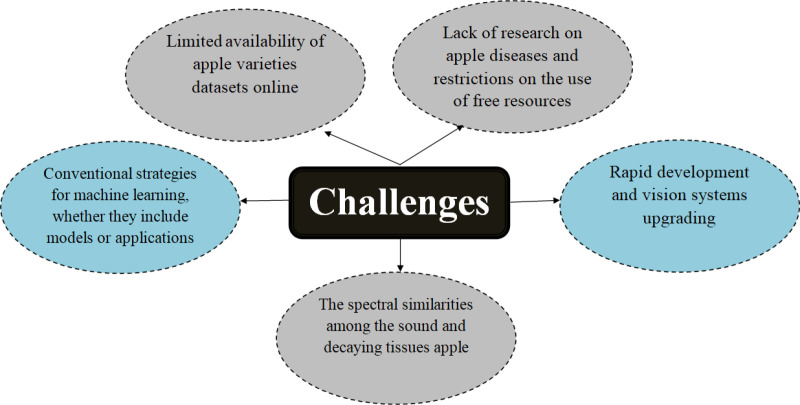
shows the difficulties and barriers facing research presently.

Regarding resources, there are many kinds of apples—up to 7,500 varieties, approximately 2,500 types are grown worldwide [https://treefruit.wsu.edu/web-article/apple-varieties]. However, there are still no publicly accessible datasets that include all the variants. Despite the rapid development of artificial intelligence technologies in apple fruit activities, the research community faces issues due to reduced accessible apple resources. Secondly, most materials on apple diseases do not cover all illnesses. Thirdly, the computerized grade system still has difficulties detecting earlier rottenness in apples because the sound and decaying tissue share considerable spectral and spatial similarities. Lastly, using traditional vision systems reduces the quality of keeping up with devices, equipment, and efficiency. Fig 1. represents the current study's challenges and obstacles.

This paper’s objectives will be as follows: First, provide free access to the resources of apple varieties online. Second, rotten fruits can harm other fresh fruits and reduce production if not correctly disposed of. Consequently, we suggest a method to decrease labor costs and times while reducing human exertion by recognizing apple faults in the agriculture industry early. Third, it is essential to understand the many illnesses that affect apples and how to manage them to prevent plant diseases from causing harm. Thus, creating an automated system for illness diagnosis will be efficient. A disease may be quickly diagnosed and controlled, and ultimately, the loss will be lessened via the detection of samples, their categorization using image processing methods, and the neural network’s extraction of features like color and texture. Fourth, following the state of the art, we offer a new method that benefits the apple vision system, which improves the detection technique and includes the navigation feature to accomplish ongoing self-harvesting in the future. Last, traditional deep learning strategies can be upgraded by adding new and advanced modules that positively affect performance, whether with models or applications.

**Fig 2:**
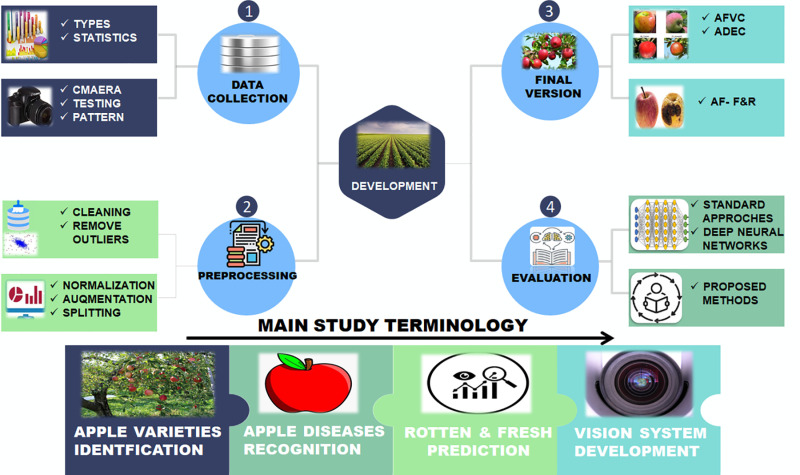
Overall description of work.

The key contributions of this paper are listed below (see Fig 2):

- Provide a comprehensive apple dataset called Apple Fruit Varieties Collection (AFVC) with 85 classes through websites and orchards, with free access [https://github.com/mustafa20999/AFVC].
- We offer new Apple Diseases Extensive Collection (ADEC) resources [https://github.com/mustafa20999/ADEC], which aid in identifying diseases and shield crops from losses.
- Present initial benchmark evaluations of the apple varieties and disease identification, besides distinguishing between fresh and rotten apples via Apple Fruit Quality Categorization [https://github.com/mustafa20999/AFQC] (AFQC).
- Develop an Optimized Apple Orchard Model (OAOM) based on the vision transformer (VT) method and a novel loss function (MFCE) that optimizes the identification tasks and model performance.
- The proposed technique outperformed standard algorithms regarding speed, efficiency, and effectiveness. Besides, it provides the highest efficiency of performance in comparison to the existing baselines.

The rest of this paper is organized as follows: Section 2 materials and methods contain the data collection, preprocessing, and statistics. Followed by the methodology includes the baseline methods utilized, the proposed method description, and the experiment setup. Section 3 results and discussion. Section 4 highlights the limitations and future research. Section 5 provides a conclusion and recommendations for further study.

## 2. Materials and Method

### 2.1. Data Collection

Images are being studied to enhance data quality or solve real-world challenges. Image categorization has been widely explored in computer vision, which involves computers comprehending digital pictures or videos. This has led to many improvements and practical applications.

**Fig 3:**
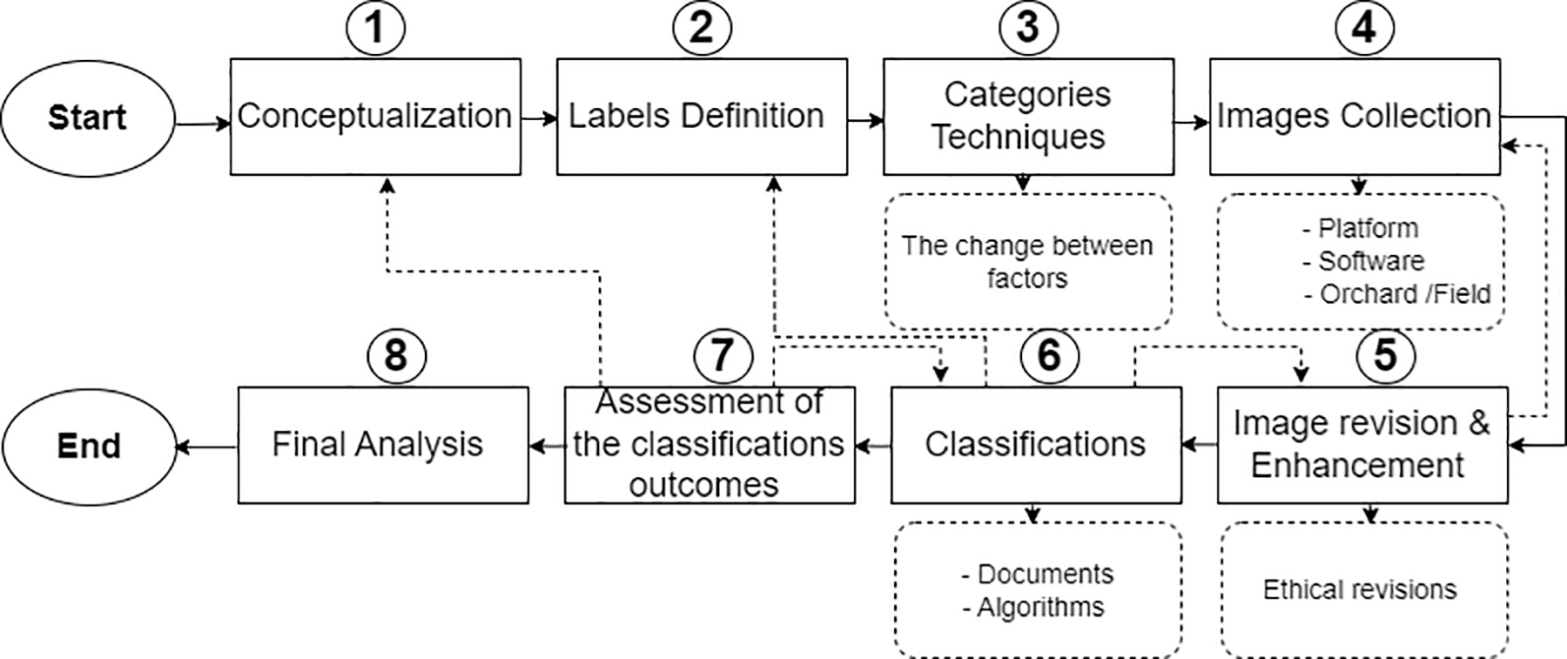
Illustrate the overall standard steps of data collection and analysis procedures.

Defining and implementing topics of interest is akin to measuring them using typical survey questions. However, picture collection and analysis are more complex than the survey inquiries. Additionally, researchers must select how to extract data from images. How researchers handle photos affects their ability to enhance data accuracy and get fresh perspectives. The steps described below (Fig 3) represent the standard steps we have implemented in the overall data collection and analysis process.

The first phase involves defining and operationalizing key ideas to ensure validity and reduce measurement mistakes. For instance, we chose each class’s apple types, damages, and illnesses. In the second step, select item labels. For the apple varieties, how many types do we have, the source of the data, and the name of each label. as we have in the apple diseases and the apple damages. In the third step, we should investigate task aspects. Manual categorization is recommended for low picture counts (around 350) due to its excellent accuracy and consistency while requiring little resources. When a trained technique is available, automated categorization is more cost-effective and faster for huge picture volumes.

Selection criteria for classification techniques. Task features include total photos, labels, label types, human resources, infrastructure, money, time, and image availability and data quality objectives such as correctness, consistency, security, and transparency. In the fourth stage, the platforms that applied and assisted in creating the data—tools, cameras, and software implemented and utilized in the collection—are included. In the fifth step, image evaluation may be critical for visual quality and ethical reasons after collection. Inadequate illumination, complicated backdrops, conflicting spots, shadows, and fuzzy photos may hinder accurate categorization. To increase the quality of images, it may be necessary to crop out unnecessary information and avoid material overlap. Apply improvements to images before categorization as necessary.

Step 6 categorizes photos based on the decision(s) taken in step 3 after ensuring image quality and removing noise. It should test the trained algorithm to discover faults and give uniform correction criteria based on the assessment approach. If difficulty is encountered in specifying the objects to be categorized, step 1 may need to be revisited. Due to faults in step 5, some low-quality photos may remain in the pool for classification. Return to step 5 to examine and improve these photographs for categorization. If enhancing their quality is complex, gather them again or omit them from the analysis. In step 7, the classification outcomes, the finding matches labels, the algorithm’s performance, and their impact on the classification are verified.

Finally, in step 8, after checking the data, univariate analysis may be performed to determine the frequencies of each label, the number of labels per picture, and other indications that may assist in extracting key essential characteristics.

**Fig 4:**
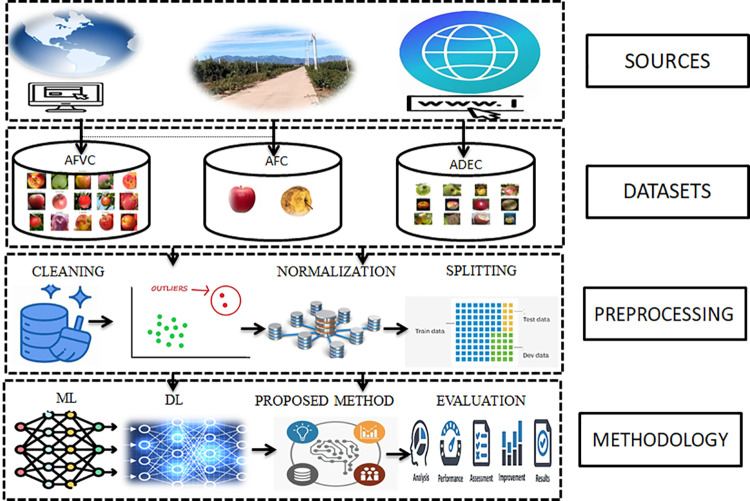
Overall description of data acquisition sources, dataset types, preprocessing, methodology, and evaluation.

This work created three datasets from various resources: orchards, global official agricultural institutions [https://nwhort.org/industry-facts/apple-fact-sheet/; https://www.foodandwine.com/types-of-apples-7976165], and the Internet [https://minnetonkaorchards.com/apple-varieties/]. Next, the preprocessing stages started with cleaning, removing the outliers, normalization, down-sampling, and splitting. After that, using deep learning models for initial assessment, the proposed method with novel loss function improves performance and quality (see Fig 4).

**Fig 5:**
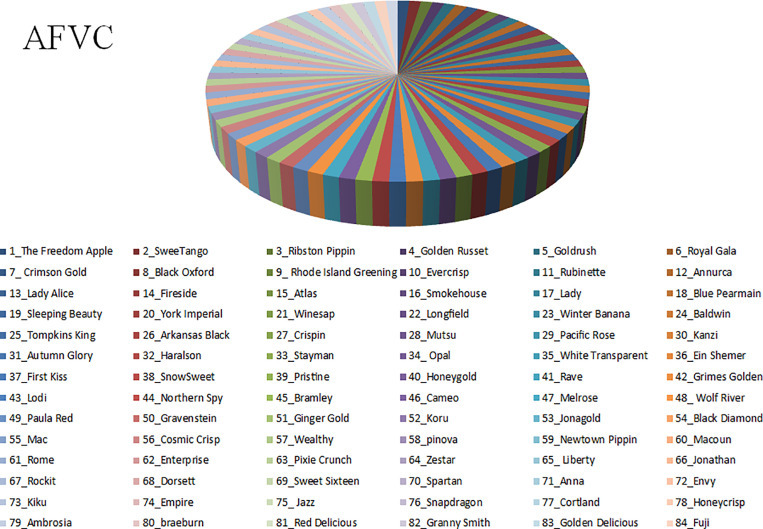
The Apple Fruit Varieties Collection (AFVC) distributions through 85 classes.

**Fig 6:**
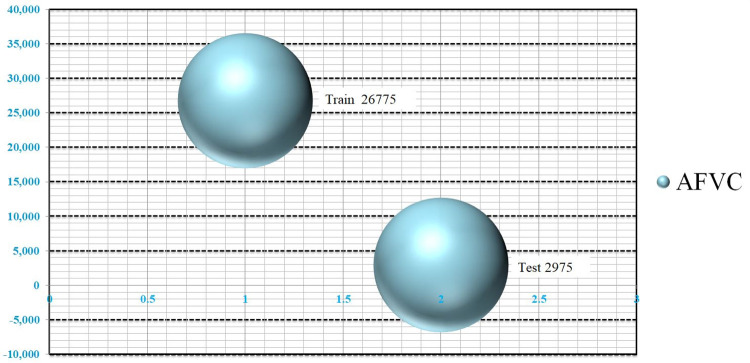
The Apple Fruit Varieties Collection (AFVC) measurement was split through 85 classes; the overall training was 26,775, and the testing was 2,975 samples.

The first grouping called Apple Fruit Varieties Collection (AFVC)(85), which consist with 85 classes, ‘The Freedom Apple’, ‘SweeTango’, ‘Ribston’ ‘Pippin’, ‘Golden Russet’, ‘Goldrush’, ‘Royal Gala’, ‘Crimson Gold’, ‘Black Oxford’, ‘Rhode Island Greening’, ‘Evercrisp’, ‘Rubinette’, ‘Annurca’, ‘Lady Alice’, ‘Fireside’, ‘Atlas’, ‘Smokehouse’, ‘Lady’, ‘Blue Pear-main’, ‘Sleeping Beauty’, ‘York Imperial’, ‘Winesap’, ‘Long-field’, ‘Winter Banana’, ‘Baldwin’, ‘Tompkins King’, ‘Arkansas Black’, ‘Crispin’, ‘Mutsu’, ‘Pacific Rose’, ‘Kanzi’, ‘Autumn Glory’, ‘Haralson’, ‘Stayman’, ‘Opal’, ‘White Transparent’, ‘Ein She-mer’, ‘First Kiss’, ‘SnowSweet’, ‘Pristine’, ‘Honeygold, Rave’, ‘Grimes Golden’, ‘Lodi’, ‘Northern Spy, Bramley’, ‘Cameo’, ‘Melrose’, ‘Wolf River’, ‘Paula Red’, ‘Gravenstein’, ‘Ginger Gold’, ‘Koru’, ‘Jonagold’, ‘Black Diamond’, ‘Mac’, ‘Cosmic Crisp’, ‘Wealthy’, ‘pinova’, ‘Newtown Pippin’, ‘Macoun’, ‘Rome’, ‘Enterprise’, ‘Pixie Crunch’, ‘Zestar’, ‘Liberty’, ‘Jonathan’, ‘Rockit’, ‘Dorsett’, ‘Sweet Sixteen’, ‘Spartan’, ‘Anna’, ‘Envy’, ‘Kiku’, ‘Empire’, ‘Jazz’, ‘Snapdragon’, ‘Cortland’, ‘Honeycrisp’, ‘Ambrosia’, ‘braeburn’, ‘Red Delicious’, ‘Granny Smith’, ‘Golden Delicious’, ‘Fuji’, and ‘Gala’. with 29,750 as shown in Table 1, and (Fig 5, Fig 6) representing the data statistics and splitting.

**Table 1:** Apple’s datasets distributions.

Datasets	Train	Test	Total
AFVC (85)	315 For each class	35 For each class	29,750
AFQC (2)	1,044 For each class	1,16 For each class	2,320
ADEC (7)	2,682	294	2,976

**Fig 7:**
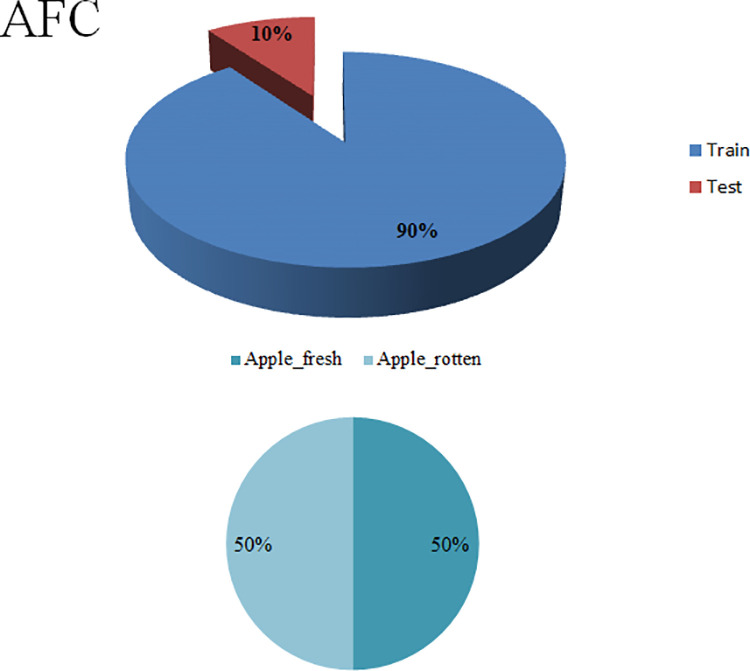
The Apple Fruit Quality Categorization (AFQC).

The second collection, Apple Fruit Quality Categorization (AFQC) [https://github.com/mustafa20999/AFQC], was collected from the orchard (Table 1). The study area was Beijing City, Huairou District, Beijing Shengshiguowang, with a mean temperature of 76°C − 19°C and an average monthly rainfall of 51.2 mm. Data was collected at two different periods between October 1st, 2023, and October 10th, 2023: in the morning, when shooting time started at 6:30 AM and finished at 11:00 AM, and in the afternoon, when shooting time started at 1:00 PM and concluded at 6:00 PM. The Nikon and Intel RealSense d435i were utilized to acquire the data. The initial total was 3,131, with dimensions of 3696 x 2448 pixels, after preprocessing, cleaning, removing outliers, cropping, normalization, dimensional reduction, and splitting. The final total has 2,320 apples in two classes: 1,160 fresh and 1,160 rotten, with 800 x 600 pixel dimensions (Fig 7).

**Fig 8:**
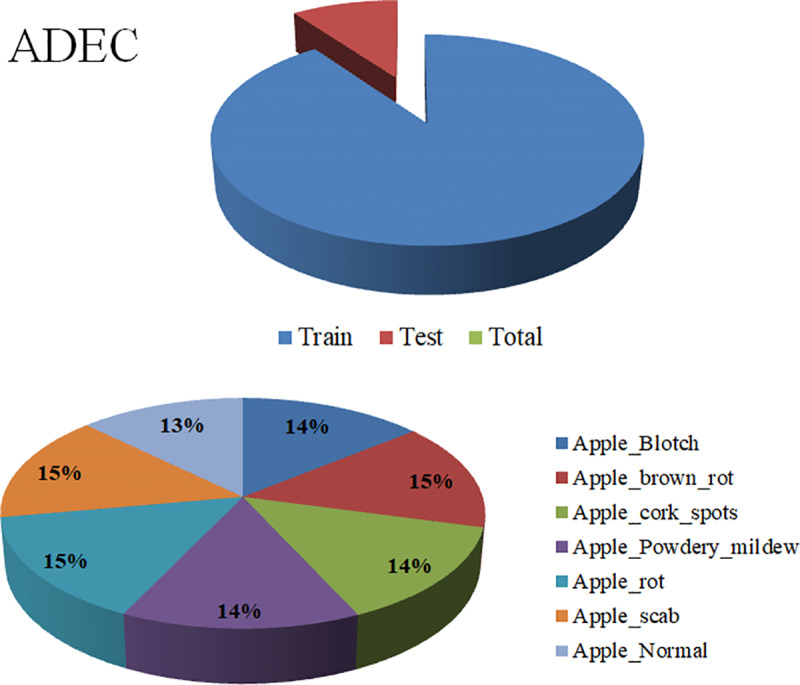
The Apple Diseases Extensive Collection distributions with fresh and rotten apples. (ADEC) distributions with seven classes.

The third assemblage, Apple Diseases Extensive Collection (ADEC) [https://github.com/mustafa20999/ADEC] resources, has a total of 2,976 and is comprised of seven classes: ‘Apple Blotch’, ‘Apple brown rot’, ‘Apple cork spots’, ‘Apple Powdery mildew’, ‘Apple rot’, ‘Apple scab’, and ‘Apple Normal’, with 90% for training, 10% for testing (Table 1). Fig 8 shows the data distributions and splits.

### 2.2. Preprocessing

Image preprocessing is converting unprocessed picture data into a format that can be used and understood. Here, we implemented several steps, starting with cleaning, which enabled us to remove undesirable distortions and improve certain aspects crucial to the image. Next, eliminating outliers lowers noise and increases analytical precision, but can also decrease sample size and introduce bias or data loss. After that, normalization stretches to normalize the intensity value range. Finally, downsamples reduces the amount of space required for the image to be delivered and stored, and it additionally decrease the dimensionality of the pictures to facilitate quicker data processing.

### 2.3. Deep Learning Models

Neural networks are a type of algorithm used in scientific problem-solving; they serve as a form of fundamental simulation of actual brain processes; learning ability is one of the primary and essential characteristics of artificial neural networks [56]. Learning and training include identifying patterns and assigning the appropriate answers [57]. Theoretically, a network should respond appropriately to inputs not used during the training and learning stages [58]. The apple-collecting dataset was analyzed using several CNN-based deep neural network designs. CNN’s low relational inductive bias and invariance under spatial translations make them a popular choice for computer vision problems [59,60].

In addition to CNN, ten models were chosen as baselines for the first assessment and analysis of the new apple collections. The models were VGG16, VGG19, CNN-LSTM, Inception V3, TF-Inception V3, VT, ResNet50, ResNet152V2, MobileNet, and EfficientNet.

### 2.4. Feature extraction

The process of converting unprocessed data into numerical features that may be handled while keeping the information in the original data set is known as feature extraction. It produces superior outcomes compared to simply implementing neural networks on the raw data [61–63]. The purpose of extracting the features in this study using deep neural networks has multiple explanations: First, reducing computational operations and execution time. Second, enhancing efficiency: algorithms frequently function more smoothly if fewer features are used. This is because the algorithm can concentrate on the most crucial elements of the input, as noise and unimportant information are eliminated. Third, better data understanding is needed to avoid overfitting.

### 2.5. Baselines

#### 2.5.1. CNN.

It consists of three convolution blocks, one max pooling layer each, which comprise the Keras sequentially architecture. A ReLU activation function activates a fully connected layer with 128 units on top of [https://colab.research.google.com/github/tensorflow/docs/blob/master/site/en/tutorials/images/classification.ipynb#scrollTo=WcUTyDOPKucd] [59].

#### 2.5.2. CNN-LSTM.

In the CNN-LSTM architecture, the LSTM handles a lengthy sequence more quickly and provides the final prediction, while the convolutional layers gather local characteristics from the input. In this case, the structure comprises an LSTM with 100 outputs, two max-poolings of size two, two convolutions with 64 and 32 filters, a flattened layer, and a dense layer [https://www.kaggle.com/code/vighneshanand/fork-of-hybrid-simple-cnn-lstm] [64].

#### 2.5.3. Inception V3.

The computational framework consists of convolutions, average pooling, max pooling, concatenations, dropouts, and fully connected layers, among other symmetric and asymmetric building pieces. The model makes substantial use of batch normalization, which is applied to activation inputs. Softmax is used to calculate the loss [65].

#### 2.5.4. TF-Inception V3.

Transfer learning (TF), which involves using a previously learned model on a new model to shorten training times and improve performance, has grown in popularity [https://colab.research.google.com/github/MahshidAlimi/ComputerVision/blob/master/Smile_Classification_using_Inception_V3.ipynb].

#### 2.5.5. VGG16.

A neural network model created for the 2014 ImageNet competition uses CNN layers [https://github.com/sbouslama/Image-classification-using-CNN-Vgg16-keras] [66].

#### 2.5.6. VGG19.

Utilizing the concept of a CNN with 19 layers, the VGG19 model uses a 224 × 224 input target size [67].

#### 2.5.7. ResNet50.

A residual neural network containing a 50-layer CNN is one kind of ANN that builds a network by piling residual blocks on top of one another [68].

#### 2.5.8. ResNet152V2.

More 3-layer blocks are used to construct large residual networks like ResNet152. Additionally, compared to VGG-16 or VGG-19 nets (15.3/19.6bn FLOPS) and even at deeper network depth, the 152-layer ResNet has much less complication (11.3bn FLOPS) [https://keras.io/api/applications/resnet/#resnet152v2-function].

#### 2.5.9. MobileNet.

It employs the principles of the point-wise convolutional technique and the depth-wise convolutional approach. Kera’s framework was used to invoke the MobileNet algorithm [69].

#### 2.5.10. EfficientNet.

It’s a CNN design and scaling technique that uses a ‘compound coefficient’ to equally scale all dimensions of depth, breadth, and resolution [70].

#### 2.5.11. Vision Transformers (VT).

The architecture of a transformer, first created for text-based activities, served as the foundation for the visual model known as ViT. The ViT approach anticipates labels of classes for an input picture by treating it as a sequence of image patches, similar to the word embedding’s used when text is altered using transformers [https://keras.io/examples/vision/image_classification_with_vision_transformer/] [71].

### 2.6. Proposed Method

#### 2.6.1. OAOM Architecture.

**Fig 9:**
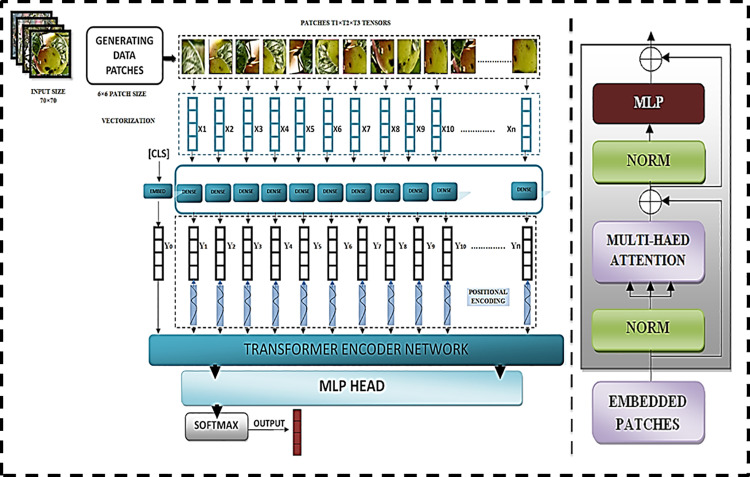
The proposed optimized Apple orchard model vision transformers architecture, patches, vectorizations, Transformer Encoder Network, and MLP head.

The proposed optimized Apple orchard model vision transformer (OAOM-VT) (Fig 9) is optimized based on the vision transformer (VT) method [71]. The main components of OAOM consist of twelve layers, hidden size (D) was seven hundred ninety, MLP size up to three thousand ninety-two, and the number of the Heads (H) were twelve, advanced regularization functions that assist in reducing overfitting, and the proposed measured focal cross-entropy (MFCE) which helps in improving the proposed model’s efficiency that contributes to raising the efficiency of the suggested strategy. We will now describe the proposed architecture and the optimization via each phase from the input layer to the final output. In terms of the model’s input, we first create our input shape, beginning with a 70 × 70 picture and a patch size of 6 × 6. To avoid non-overlapping of the input patches, we use the same stride of patches (6 × 6), which lessens the computing cost of figuring out how many pixels travel over the window each time.

Next, ascertain which patches per picture (N) correspond to (H × W)(Ph × Pw). W stands for weight, and H for the high image: patch height, patch weight, Ph, and Pw. The element per patch (Ep) is next. T_1_ × T_2_ × T_3_ tensors were the stages. Second, during the vectorization step, restructure the T_1_ × T_2_ × T_3_ tensors to create X_1_, X_2_, X_3_,..., X_n_-dimensional vectors. Next, using the following equations as a basis, apply a linear function to the dimensional vector output.


$$\begin{array}{c} Y_{1}=wA_{1}+b \end{array}$$ (1)

$$\begin{array}{c} Y_{2}=wA_{2}+b \end{array}$$ (2)

$$\begin{array}{c} Y_{N}=wA_{N}+b \end{array}$$ (3)

W represents the matrix parameters from training data, and A vector is a parameter to be learned. And b from training data, a dense layer for sharing parameters. In addition, positional encoding was added to the vectors Y_1_, Y_2_, and Y_n_. Each patch has a position and an integer between 1 and n.

Positional encoding converts an integer into a vector whose form is identical to Y. Side to P_n_ patches: the embedding layer receives the output vector Y_0_ input and the CLS token as prediction and classification input. Y_0_ has the same form as vectors two and three. Following that, it provided further details on the transformer encoder networks (TEN) and the CLS token fitting sequence 0 to Y_n_ (Fig 9A). Switcher Following the multi-head attention layer for processing and managing the input sequence, skip connection and normalization and go on to the MLP Head for the final optimization. Softmax is then used to forecast the desired output with multiple dimensions.

#### 2.6.2. TEN.

The transformer encoder comprises multi-head self-attention (MHSA) and layer norm (IN) [72], applied before every block, and residual connection after every block [73]. Different input sequence segments may be handled in various ways due to MHSA. Each head will pay attention to a separate input element independently, allowing the structure to catch features better and increase pattern representations. The following formulas define the MHSA:


MultiHead(Q,K,V=[head1,......headh]W0 (4)

headi=Attention(QWi,KWi,VWi) (5)

W represents all the learned parameters in the matrix presented above. The optimization of the TEN (Fig 9B), based on the architecture proposed by Alexey Dosovitskiy [71], involves reducing the total number of parameters, hidden size, and MLP. Additionally, we provide the optimal hyperparameter adjustment regarding input and expected outputs via various dimensions (see **2.7.)**. TEN received the outcome sequence of the vectors (OSV), then processed (P) through layers, the encoder block (EB), and the transform block (TB) to provide them MLP HEAD, based on the below equation:


TEN=OSV*P(EB,TB) (6)

#### 2.6.3. Loss Function.

A machine learning statistic called cross-entropy loss assesses how effectively a categorization model works. A value between 0 and 1 represents the loss (or error), with 0 being flawless modeling. Cross-entropy (CE) is a common loss function widely used in a range of categorization issues. The following equation represents key points of (CE):


$$\begin{array}{c} CE(Z,EP)=\sum_{j=1}^{m}=1Zj\log(EPs) \end{array}$$ (7)

Where Z represents the actual probability, and EP represents the estimated probability. In particular, the probability related to ground truth for a one-hot encoding scheme is one. If not, it is zero. Even with unbalanced data, the cross-entropy loss function often performs poorly. Big classes possess a higher loss cost than total training loss during the model training phase, which biases the model in favour of significant categories. Conversely, classification efficiency could be better for categories with less data.


$$F(Z,EP)=-\sum_{j=1}^{m}\;Z_{j}{\left(1-EP_{j}\right)}^{\gamma }log\left(EP_{j}\right)=-{\left(1-EP_{s}\right)}^{\gamma }log\left(EP_{s}\right)$$ (8)

A targeted loss function was introduced by Lin et al. [74] to force the classifier to focus more on poorly categorized data sets. The targeted loss (8) assigns the samples different weights according to estimated probability.

Here, the hyper-parameter γ is adjustable. The focused loss will shift to cross-entropy loss when γ equals zero. In other situations, focus loss has an extra cost compared to cross-entropy. As the value of γ rises, the penalty also grows exponentially. Overall, models with a large quantity of content can learn enough information about features from classes. The machine learning algorithm already properly classifies these samples, so focus loss will give them a lower weight. In addition, the model has trouble correctly classifying minor categories due to insufficient training data.

This negative effect might be lessened if a targeted loss gave poorly recognized samples a notably more significant weight to draw the method’s attention. Because of this, these samples nonetheless make up a substantial portion of the total training loss, even though they are few. However, focal loss could be better. More specifically, for small projected probability, primarily for large γ, the concentrated loss’s loss value will be much weaker than the cross-entropy’s.

We suggest a new loss function, called measured focal cross-entropy (MFCE) loss, with a configurable weight parameter tw ≥ zero, which is defined as (9), taking into account the constraint above


$$MFCE=-\sum_{j=1}^{m}\;Z_{j}[\frac{e^{{\left(1-EP_{j}\right)}^{d}v}}{(e^{E}P_{j}+e^{{\left(1-EP_{j}\right)}^{d}v)}}log\left(EP_{j}\right)+\frac{e^{{\left(EP_{j}\right)}^{d}v{\left(1-EP_{j}\right)}^{\gamma }}}{\left(e^{E}P_{j}^{d}v+e^{{\left(1-EP_{j}\right)}^{d}v}\right)}log\left(EP_{j}\right)]$$ (9)

$$\frac{e^{{\left(1-EP_{s}\right)}^{d}v}}{(e^{E}P_{s}^{d}v+e^{{\left(1-EP_{s}\right)}^{d}v)}}\log\left(EP_{s}\right)-\frac{e^{{\left(EP_{s}\right)}^{d}v{\left(1-EP_{s}\right)}^{\gamma }}}{(e^{E}P_{s}^{d}v+e^{{\left(1-EP_{s}\right)}^{d}v)}}$$

**Fig 10:**
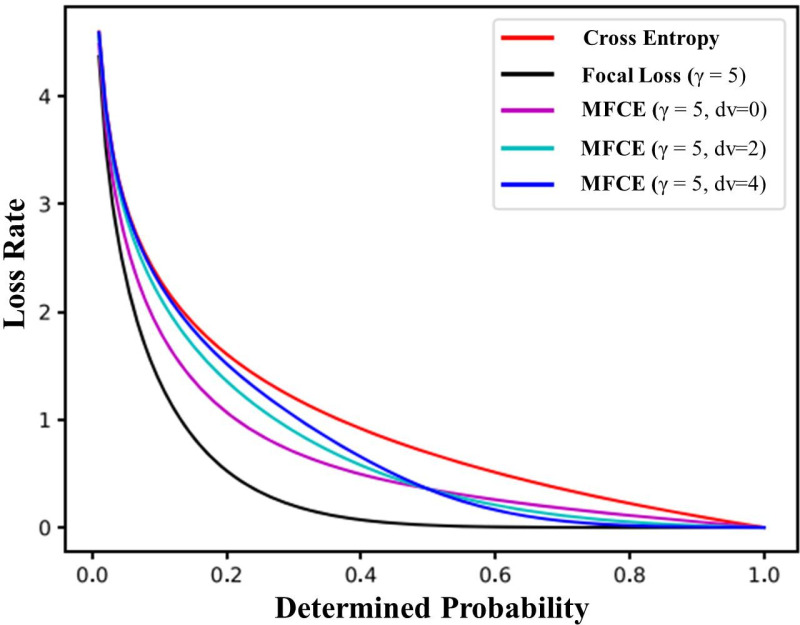
The MFCE loss function curves with.

MFCE loss function curves with various dv values are shown in Fig 10 to intuitively illustrate the suggested loss function’s qualities. Every MFCE loss curve falls between focal loss and cross-entropy. In the interim, the concentrated loss gradually approaches the CEWF loss as the expected probability increases. At estimated probabilities around zero, MFCE loss is more consistent with cross-entropy. Moreover, for larger dv values, this trend is more noticeable. Consequently, the MFCE loss of a well-classified sample will be less than the cross-entropy loss. In addition, a sample with a poor classification will suffer a loss more significant than the focal loss. Therefore, it could reduce the model’s bias in favor of large categories and improve the categorizing accuracy of minor subclasses.

**Fig 11:**
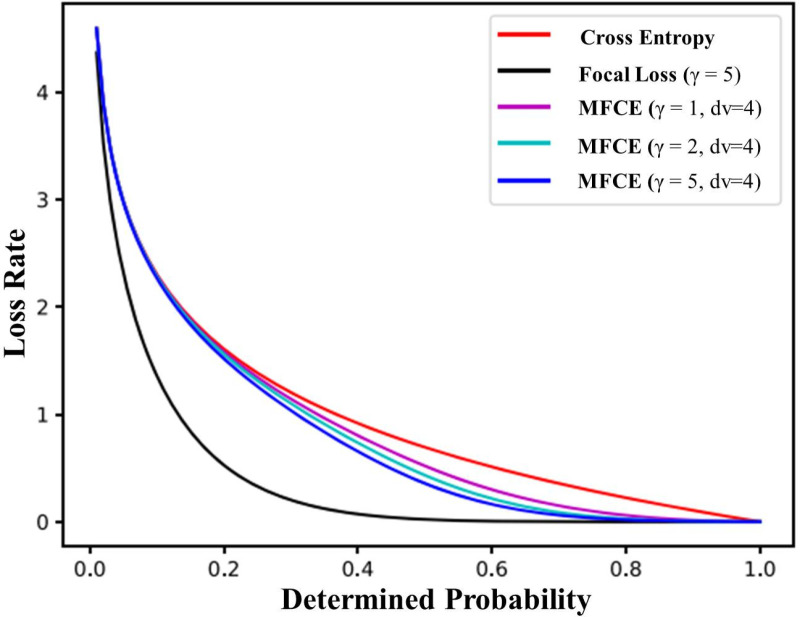
The curves of the MFCE loss function with varying values of dv.distinct γ values.

Furthermore, MFCE loss function curves with various γ values are shown in Fig 11. It is evident that for samples with poor classification, γ has a minimal effect. Moreover, as γ increases, the MFCE loss diminishes for estimated probabilities around one.

### 2.7. Experimental Settings

The framework of the technique is assessed and learned using the following metrics. Four varieties of dimensional prediction exist: False Positives (FP) [75] are false positive classifications; False Negatives (FN) [76] are false negative classifications; and True Positives (TP) [77] are the number of correctly classified positive dimensions. True Negatives (TN) [78–81] are the number of accurately classified unfavourable classes. After that, the efficiency metrics listed below are computed:


Accuracy=TP+TN÷TP+FP+FN+TN (10)

Precision=TP÷TP+FP (11)

Recall=TP+FN (12)

F1−score=2*Precision*Recall÷Precision+Recall (13)

These were the tuning and hyper parameter settings used: pooling [**2, 4, 6**, 8, 10], batch-size [16, 32, 64, 128, 164], kernel-size [**3, 5, 7**, 10, 15], number-classes [**2**, 3, 4, 5, **7**, 8, 9, **85**], epoch [**10, 20, 50, 100**, 200, 500], and Adam optimizer with 0.001 learning rate. The Keras library was implemented using Python. Table 2 lists the hardware and software setups for the experimental environment.

**Table 2:** The environment configuration for the experiment.

Configuration elements	A setup parameters
CPU	Intel(R) Core(TM) i7-10870H CPU @ 2.20GHz 2.21
GPU	GH NVIDIA GeForce RTX 4060 Ti
RAM	32GB
DEEP LEARNING FRAMEWORK	Keras, TensorFlow 2.12.
PROGRAMING LANGUAGE	Python 3.12.1

## 3. Results and discussion

### 3.1. The preliminary evaluation of the new apple collections using deep learning models

This experiment aims to initially evaluate the AFVC (85) dataset to distinguish the apple varieties, the AFQC (2) dataset to recognize the fresh and rotten apples, and the ADEC (7) dataset for apple disease identification. Standard deep learning approaches are CNN, CNN-LSTM, InceptionV3, and transfer learning of InceptionV3.

In addition to enhancing the core architectures with optimal hyperparameter choices, we added additional filters to our improved CNN model and Inception V3. OP-CNN may use several window weights and lengths depending on the number of feature maps needed to be produced. Several building components and operations that comprise the layer’s output were optimized, and the appropriate activation function was given for each activity.

The configuration of DL models was some classes equal to three; the split (Train/Validation/Test) was 80%/10%/10%, and the kernel size was three or five, with various pooling between two and four, batch-size [64, 128], Adam optimizer, 0.001 learning rate, and one hundred epochs.

On AFVC (85) datasets, the model performance was (55.42%) for CNN, (71.02%) for the optimized CNN, (72.55%) for CNN-LSTM, (74.13%) for InceptionV3, and (77.34) for pertained InceptionV3 (Table 3).

**Table 3:** The accuracy (%) of five deep-learning models on the AFVC, ADEC, and AFQC datasets.

Models	AFVC (85)	ADEC (7)	AFQC (2)
CNN	55.42	74.06	90.89
OP-CNN	71.02	91.23	92.57
CNN-LSTM	72.55	73.81	92.42
InceptionV3	74.13	94.65	96.12
InceptionV3-TL	**77.34**	**95.83**	**98.44**

On ADEC (7) datasets, the model performance was (74.63%) for CNN, (91.23%) for the optimized CNN, (73.81%) for CNN-LSTM, (94.65%) for InceptionV3, and (95.83%) for pertained InceptionV3.

For AFQC (2) datasets, the model performance was 90.89%, 92.57%, 92.24%, 96.12%, and 97.44%, respectively.

**Fig 12:**
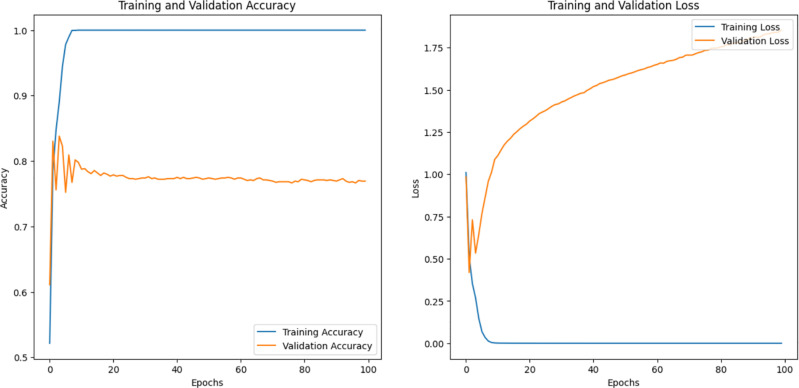
Accuracy, Loss, and validations of the pertained InceptionV3 on AFVC (85) dataset.

**Fig 13:**
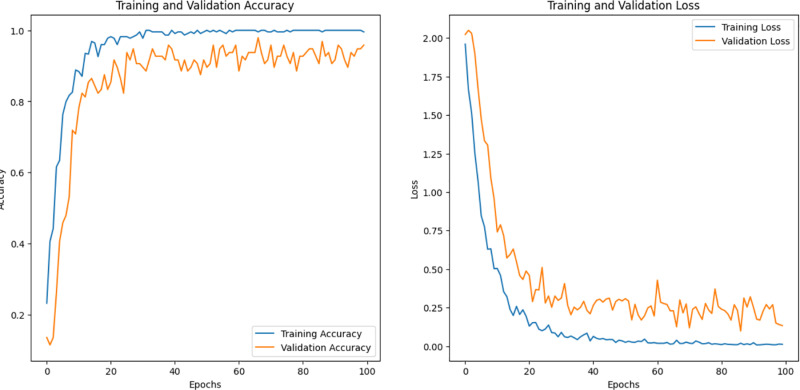
Accuracy, Loss, and validations of the pertained InceptionV3 on ADEC (7) dataset.

**Fig 14:**
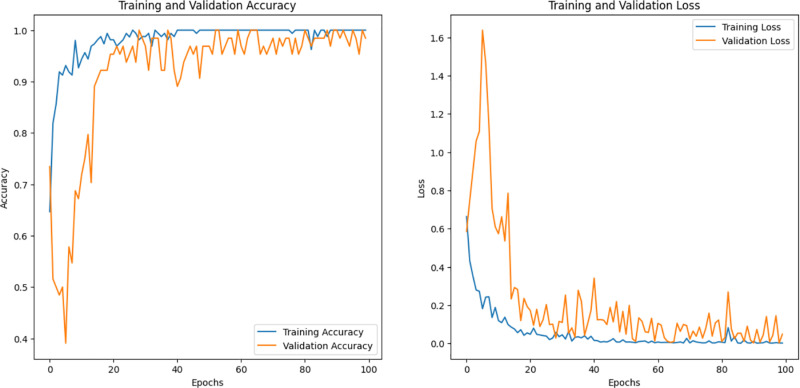
Accuracy, Loss, and validations of the pertained InceptionV3 on AFQC (2) datasets.

We note that pertained InceptionV3 and optimized CNN performed best among the other models; pertained InceptionV3 had the highest efficiency, for AFVC (85) accuracy was 77.34% (Fig 12), for ADEC (7) was 95.83% (Fig 13), and for AFQC (2) was 98.44% (Fig 14). We see that the study that focused on the AFQC (2C) had the highest performance compared to ADEC (7C) and AFVC (85C). In comparison to the earlier studies, [40,41] use SVM to differentiate between fresh and rotten apples with 98.90% and 95.27%, respectively. The training consumes time even if it is evaluated in a small amount of the datasets. One of their approaches’ limitations is the complexity of selecting the best kernel due to the limitless amount of options available. Few kernels are utilized in most applications, and these kernels are situation-generalizable in a limited number of scenarios. It can need a lot of processing power.

The present testing is conducted on large amounts of data gathered with complicated backgrounds and various circumstances. InceptionV3 had the most successful performance. Among the current models, this is the most accurate and efficient model for processing both fresh and rotting apple photos. It can more accurately identify specific patterns, features, and images. The model can recognize dynamic items in a single frame, including visible objects. It is thus appropriate for a wide range of apple image processing applications, from real-time apple identification to damage recognition and categorization. Nevertheless, the optimized CNN performed better in terms of time. Ten-fold cross-validation was conducted for all techniques, and the average performance was provided, as shown in Table 3.

### 3.2. Deep neural networks to extract deep features from apple datasets

The purpose of the second experiment is to utilize deep neural networks to extract the best features of the AFVC, ADEC, and AFC datasets and provide an initial benchmark analysis study for differentiating between apple varieties’ features. Second, identify the apple disease. Third, recognize the fresh and rotten apples, and analyze the proposed methods of measurement and prediction.

The eight models applied were VGG16, VGG19, ResNet50, ResNet152V2, MobileNetV2, EficientNetV2B0, Vision Transformer (VT), and the proposed OAOM-VT. Once again, the split (Train/Validation/Test) was 80%/10%/10%. The Table shows the configuration and tuned hyperparameter of the implemented methods (Section 2.7).

**Fig 15:**
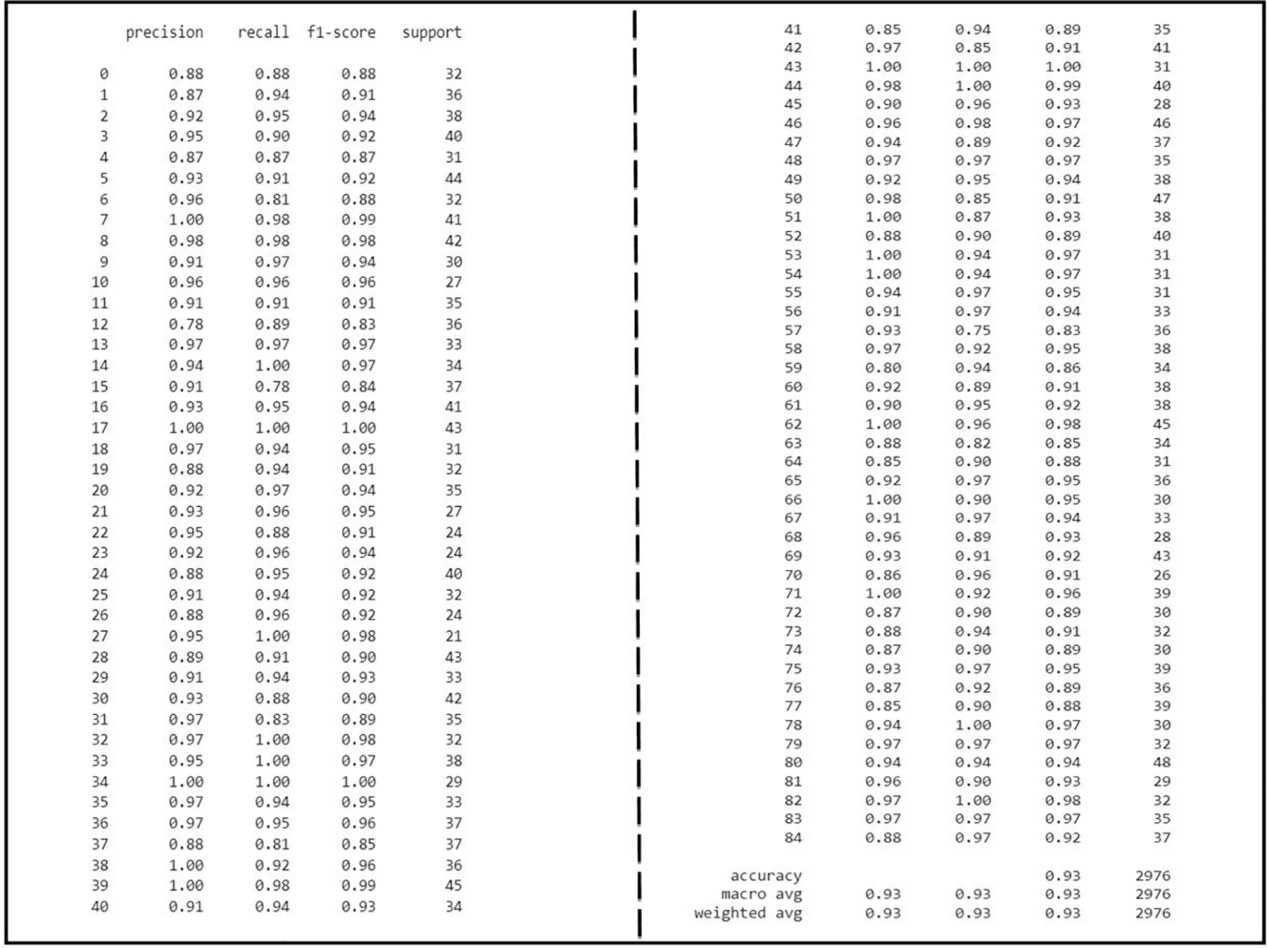
The proposed method’s accuracy, average, weight, precision, recall, and f1-score on AFVC (85).

**Fig 16:**
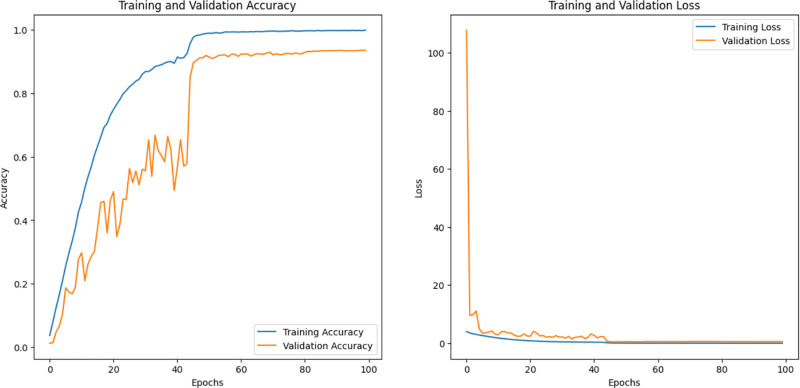
Accuracy, Loss, and validations of the OAOM-VT on AFVC (85) dataset.

We applied VGG16, VGG19, ResNet50, ResNet152V2, MobileNetV2, Ef-ficientNetV2B0, VT, and OAOM-VT on the AFVC dataset and accuracies were 61.51%, 63.98%, 68.86%, 71.94%, 75.76%, 72.42%, 88.89%, and 93.85%, respectively, as shown in Table 4. OAOM-VT architecture performed highest up to 93.85%, with a training time of (164m 57s) (Fig 15 and Fig 16).

**Table 4:** The assessment outcomes using the apple dataset sets for eight deep neural networks. Values are in percentages.

Models	AFVC (85)	ADEC (7)	AFQC (2)
VGG16	61.51	66.28	93.53
VGG19	63.98	77.21	95.59
ResNet50	68.86	82.14	93.93
ResNet152V2	71.94	93.64	97.60
MobileNetV2	75.76	96.46	96.65
EficientNetV2B0	72.42	97.28	96.12
VT	88.89	98.98	97.84
OAOM-VT	**93.85**	**99.66**	**98.28**

**Fig 17:**
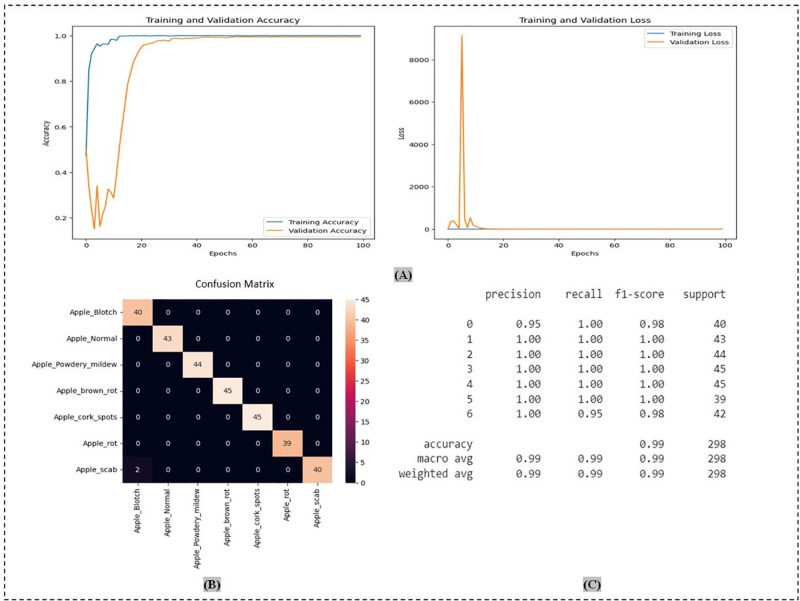
The proposed method OAOM-VT on ADEC datasets: (A) accuracy and loss validations; (B) confusion matrix; (C) precision, recall, and f1-score.

On ADEC datasets, the performance was (66.28%) for VGG16, (77.21%) for VGG19, (82.14%) for ResNet50, (93.64%) for ResNet152V2, (96.46%) for MobileNetV2, (97.28%) for EficientNetV2B0, (98.98%) for VT, and (99.66%) for the optimized (VT). Again, OAOM-VT had the highest performance, as shown in Fig 17, which illustrates the assessment measures of the proposed method.

**Fig 18:**
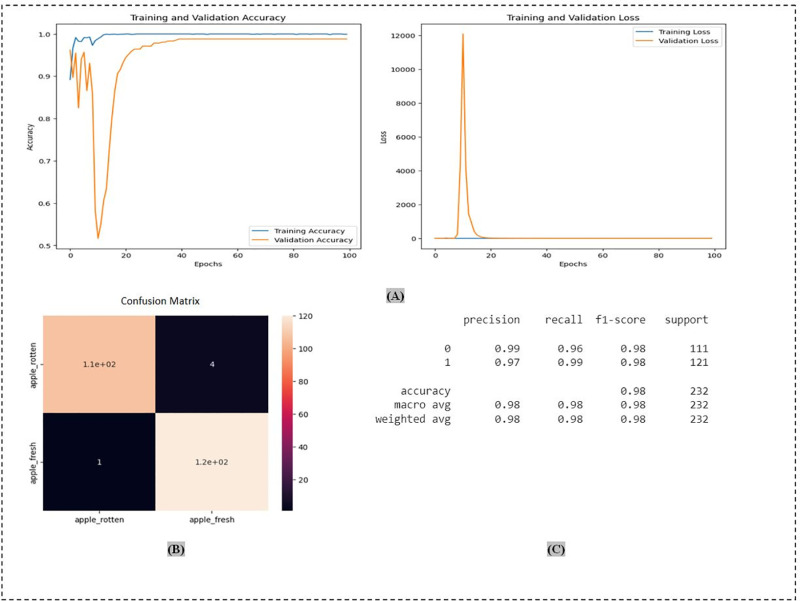
The proposed method OAOM-VT on AFQC datasets: (A) accuracy and loss validations; (B) confusion matrix; (C) precision, recall, and f1-score.

On AFQC datasets the performance VGG16, VGG19, ResNet50, ResNet152V2, MobileNetV2, Ef-ficientNetV2B0, VT, and OAOM-VT was (93.53%), (95.59%), (93.93%), (97.60%), (96.65%), (96.12\%), (97.84%), and (98.28%). The best result was achieved by OAOM-VT, as shown in Fig 18, which displays the proposed method’s evaluation metrics.

ADEC (7C) had the best accuracy (99.66%) compared to AFQC (2C) (98.28%) and AFVC (85C) (93.85%). When we compare to work related to apple diseases, previous studies [48–50] used machine learning algorithms and tested on a small amount of data, less than 500 samples, with performance of 93.00%, 95.94%, and 96.00%. For deep learning [52–55], the performance was 99.20%, 98.00%, 96.43%, and 73.70%. All the above-mentioned studies focused only on three types of diseases: scab, rot, and blotch. The present investigation implemented sophisticated neural networks. Additionally, it is more extensive and concentrated on identifying the six following diseases: Apple Blotch, Apple Brown Rot, Apple Cork Spots, Apple Powdery Mildew, Apple Rot, and Apple Scabs.

Though OAOM-VT was the most accurate model, as shown in Table 4, we observe that MobileNetV2 was the model that used less time in both datasets. OAOM-VT extracts the features of two and seven classes more efficiently than the eighty-five classes. Compared to earlier trial models, OAOM-VT is less susceptible to data augmentation and more efficient at extracting deep local features.

### 3.3. Assessment of the OAOM-VT using several standard datasets with different categories

This experiment aims to evaluate the proposed method using various existing datasets with different lengths, sizes, and classes. The first dataset includes 13599 pictures with six classes of fresh and rotting apples, bananas, and oranges [https://github.com/Bangkit-JKT2-D/fruits-fresh-rotten-classification]. Apple blotch, apple rot, apple scab, and typical apple are the four different classifications of the second one. Every class has 80 photos, with a final total of up to 320 Apple photos [https://www.kaggle.com/datasets/kaivalyashah/apple-disease-detection]. The third one used dataset at [https://www.kaggle.com/datasets/moltean/fruits/versions/22], which included apples, fruit, and vegetable classes, but only four classes were used to match the previous baseline studies.

When we implemented the proposed OAOM on the Fresh-Rotten-Fruits (FRF) (6C), Apple Fruit Disease (AFD) (5C), and Fruits-Apple Varieties (F-AV) with four classes, the performance was 99.80%, 98.23%, and 96.64% compared to 97.82%[82], 96.00%[50], and 94.35%[83], respectively, as shown in Table 5.

**Table 5:** The result of the OAOM-VT model on various fruit categorization datasets with six, five, and four classes. Values are in percentages.

Datasets	Model	Accuracy
FRF (6C)	[82] baseline	97.82%
	Proposed Method	**99.80%**
AFD (5C)	[50] baseline	96.00%
	Proposed Method	**98.23%**
F-AV (4C)	[83] baseline	94.35%
	Proposed Method	**96.64%**

**Fig 19:**
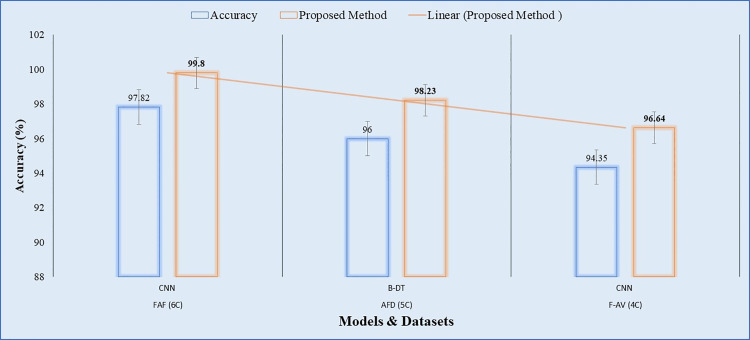
The outperforms of the proposed method OAOM-VT with baselines on FAF (6C), AFD (5C), and F-AV (4C).

In [82] and [83], they used CNN methods, which gave good accuracy as an initial baseline. However, using automated neural architecture search techniques via agriculture field and apple fruit images with low quality made it more complicated and time-consuming due to the deep hyperparameters procedure that entails various investigations. Furthermore, information loss may occur in deeper CNNs; it may be lessened by using strategies such as residual structures. When Bagged Decision Trees were used for feature extraction in [50], the performance was acceptable relative to the size of the dataset. It has a tendency to overfit the training program. When a tree is intricate and deep, it becomes overfitted and starts to identify noise in the data instead of the underlying patterns. This results in poor generalization to fresh, unobserved data. Furthermore, it produces a sequence of distinct, step-like decision boundaries, which may cause the forecasts to be less smooth. Instead of producing forecasts that follow a smooth curve, regression analysis might provide results that resemble staircases. This lack of smoothness may not be acceptable for specific uses. Still, it needed to be more optimal for higher-dimensional data. The attention methods significantly increase transformers’ suggested OAOM training speed, and our customized loss function further enhances performance. The proposed OAOM outperformed the baselines (Fig 19) and demonstrated it could work with different categories with high efficiency.

## 4. Limitations and Future research

Computer vision systems have contributed significantly to developing agricultural systems, especially in apple fruit, reducing labor effort, time, and speed in all apple operations from planting to harvesting. The current study contributed to the continued development and upgrading of the apple vision growth system, development policies, automatic sorting systems, and the decision-making process. However, to keep pace with the systems and equipment, some limitations and recommendations must be considered in the near future to upgrade performance and quality and increase production.

- It requires testing the model in real-time using a UAV or harvesting platform, for instance, to differentiate between fresh and rotten. It also involves grating with automated sorting machines and systems to see if some products, such as jam, juice, or animal food, can be recycled.
- There are still diseases not recognized in the early stages of apple growth, which assists in fast treatment. More training is needed to improve the models and enhance the identification system platform, which helps stabilize production.
- For apple varieties, there is still a chance to extend the range to 2500 types to cover the most apple types on earth. Furthermore, sophisticated satellites can be utilized for identification. This will assist in providing a robust and advanced apple sorting system in the coming years.
- With the rapid development of AI, the proposed model can be used to identify diseases or measure and detect damage in various disciplines, such as fruits, vegetables, or crops.

## 5. Conclusion

In this study, we first presented a newly comprehensive gathering called the Apple Fruit Varieties Collection (AFVC) with 29,750 images through 85 classes. After that, we differentiate between rotting and fresh apples with Apple Fruit Quality Categorization (AFQC), followed by the identification of the diseases with Apple Diseases Extensive Collection (ADEC). With 95.83% for AFVC, 97.44% for AFQC, and 95.83% for ADEC, InceptionV3-TL was the best of the initial five deep-learning benchmark evaluations. Next, an optimized Apple Orchard Model (OAOM) with a new loss function named measured focal cross-entropy (MFCE) was developed, which helped enhance the proposed model’s efficiency. The suggested OAOM performs best, with an accuracy of 93.85% for identifying apple types using AFVC. The AFQC’s rotten apple identification rate was 98.28%. The percentage of illnesses identified by ADEC was 99.66%. OAOM outperformed baselines and operated with high efficiency.

Regarding these outcomes, we have provided free access to the collections of apple resources to the research community. Furthermore, we offered and released the OAOM model, which is highly efficient for apple variety recognition, rotten apple detection, and automated apple disease identification. This proposed method benefits apple’s robotic vision advancement. It can integrate with automatic sorting and harvesting systems and upgrade their performance.
